# Commentary: The icmF3 Locus is Involved in Multiple Adaptation- and Virulence-related Characteristics in *Pseudomonas aeruginosa* PAO1

**DOI:** 10.3389/fcimb.2015.00083

**Published:** 2015-11-18

**Authors:** Le Tang, Xiaoye Liang, Richard Moore, Tao G. Dong

**Affiliations:** Ecosystem and Public Health, University of CalgaryCalgary, AB, Canada

**Keywords:** *Pseudomonas aeruginosa*, PAO1, antimicrobial activity, T6SS, IcmF, strain divergence

*Pseudomonas aeruginosa* is an important pathogen commonly isolated from patients with burns, wounds and cystic fibrosis (Lyczak et al., 2000; Gellatly and Hancock, 2013). The *P. aeruginosa* strain PAO1 was originally reported as a wound isolate from a patient in Australia in 1955 (Holloway, 1955), and has since been studied in many laboratories as a reference strain (Stover et al., 2000). However, a number of genetic variants of PAO1 in different laboratories have been reported including a large 2.2 Mb inversion and a number of single nucleotide variants and insertion-deletion mutations (Stover et al., 2000; Heurlier et al., 2005; Klockgether et al., 2010).

The type 6 secretion system (T6SS) functions as a molecular weapon that delivers toxic effectors to prokaryotic and eukaryotic target cells (Ho et al., 2014). The T6SS was first functionally characterized in *Vibrio cholerae* and *P. aeruginosa* PAO1 by the Mekalanos group in 2006 (Mougous et al., 2006; Pukatzki et al., 2006), and thereafter the PAO1 strain has been used as an important model to study the T6SS functions (Ho et al., 2014; Russell et al., 2014). PAO1 possesses three distinct T6SS clusters (H1, H2, and H3) of which the H1-T6SS delivers six known antimicrobial substrates (Hood et al., 2010; Whitney et al., 2014). The H2- and the H3-T6SS are implicated in both antimicrobial and anti-eukaryotic activities and can secrete PldA and PldB phospholipases, respectively (Lesic et al., 2009; Sana et al., 2012; Russell et al., 2013; Jiang et al., 2014).

The T6SS main structure consists of an outer sheath, an inner tube, and a membrane-bound anchor complex (Basler, 2015). Contraction of the outer sheath ejects the inner tube and its associated effector proteins to the extracellular environment (Ho et al., 2014; Basler, 2015). IcmF, a key T6SS protein, carries an ATPase domain (Ma et al., 2012) and interacts with TssL and TssJ to form a membrane-spanning complex with a hollow space that allows the inner tube and effectors to travel through (Basler, 2015; Durand et al., 2015). The recently published paper by Lin et al. (Lin et al., 2015) reported multiple interesting yet moderate phenotypes associated with the IcmF3 of the H3-T6SS, including iron acquisition, bacterial killing, motility, antibiotic resistance, and virulence. However, the molecular mechanism of IcmF3 involvement in such diverse cellular processes is not clear. Because IcmF is required for T6SS assembly, those reported phenotypes suggest a broad versatile role of the H3-T6SS in PAO1 (Lin et al., 2015). Interestingly, the T6SS in *Yersinia pseudotuberculosis* is involved in Zn^2+^ transportation (Wang et al., 2015). An alternative but not mutually exclusive explanation is that IcmF3 itself regulates other cellular processes in addition to its primary role as a key T6SS component.

Lin et al. tested *Escherichia coli* survival after co-incubation with PAO1 or the *icmF*3 mutant for 36 h and reported that the CFU of *E. coli* remained at a high level (10^8^ CFU/ml, see Table S3 in Lin et al., 2015). We found this surprising because the PAO1 strain in our lab (L-PAO1), obtained from J. Mekalanos (Mougous et al., 2006; Basler et al., 2013) and originally from S. Lory, can efficiently kill *E. coli* cells after 24 h co-incubation. Considering the reported genome divergence of PAO1 in different laboratories (Klockgether et al., 2010), we hypothesized that different PAO1 sublines may have variable killing abilities against *E. coli*. To test this hypothesis, we carried out *E. coli* killing assays using several PAO1 strains from different sources (Figure 1A), of which the L-PAO1 has been used extensively in T6SS research (Mougous et al., 2006; Hood et al., 2010; Basler et al., 2013; Ho et al., 2013). The H-PAO1 and the M-PAO1 are isolates from R. Hancock and C. Manoil, respectively, and are the host strains for two defined PAO1 transposon mutant libraries (Jacobs et al., 2003; Lewenza et al., 2005). The P-PAO1 strain and the V-PAO1 strain are from M. Parsek (Colvin et al., 2011) and E. Banin (Cohen et al., 2015), respectively. We followed the reported protocol by Lin et al. (2015) with minor modifications, primarily that the killing was done on LB medium directly instead of a filter membrane. Overnight cultures of PAO1 and *E. coli* MG1655 carrying a pPSV37 plasmid vector (gentamycin resistance) (Lee et al., 2010) were washed with fresh LB and then mixed at a 10 to 1 ratio, followed by co-incubation on a LB agar plate at 37°C for 36 h. Survival of *E. coli* was enumerated by serial dilutions on LB-gentamycin plates. Our results demonstrate that L-PAO1, V-PAO1, and H-PAO1 eliminated *E. coli* MG1655 after co-incubation, whereas P-PAO1 and M-PAO1 had little killing activity against *E. coli* (Figures 1B,C). To test if the T6SS is required for the observed killing, we constructed the *tssB1* deletion mutant (ΔH1-T6SS) and the *tssB*1-3 triple deletion mutant (ΔT6SS) in the L-PAO1 strain. Our results show that both mutants killed *E. coli* efficiently, suggesting L-PAO1 possesses other antimicrobial mechanisms independent of the T6SS clusters.

**Figure 1:**
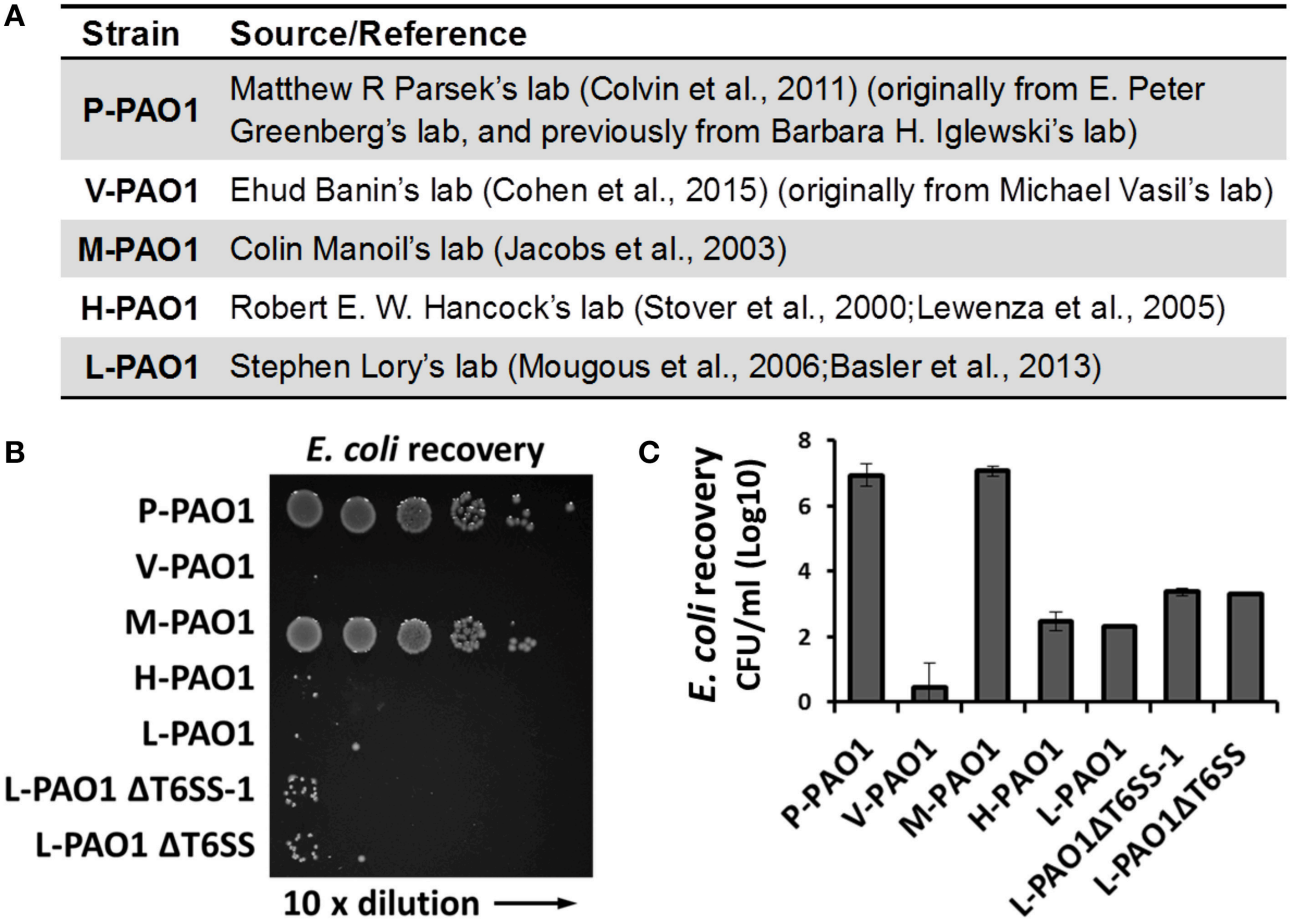
**Survival of ***E. coli*** MG1655 cells after incubation with different ***Pseudomonas aeruginosa*** PAO1 strains**. **(A)** The source of strains used in this study. **(B)** Survival of *E. coli* MG1655 pPSV37 enumerated by serial dilutions. L-PAO1ΔT6SS-1 is the *tssB*1 mutant defective in the H1-T6SS, and L-PAO1ΔT6SS is the *tssB*1-3 triple mutant defective in all three T6SS systems. **(C)** Summary of the killing assays. Experiments were performed three times. The mean values and the standard errors are shown.

The genome divergence of different PAO1 strains is known to cause phenotypic variations in virulence (Preston et al., 1995; Klockgether et al., 2010). Here we show different PAO1 strains also differ in their capability of killing neighboring cells. In complex multispecies environments such as the cystic fibrosis patient's lung, it is conceivable that competition between different species may select for PAO1 mutants with enhanced killing abilities. However, how PAO1 strains during lab passage diverge to gain/lose antimicrobial properties is not intuitively apparent. Nonetheless, because PAO1 is widely used as a model strain, researchers should be aware of the strain variations and should provide detailed description of the strain source to allow the *P. aeruginosa* community to better interpret the results.

## Author contributions

LT, XL, RM performed the experiments. TD conceived the study and designed the experiment. LT and TD wrote the paper.

## Funding statement

This work was supported by a start-up grant of the University of Calgary and a Canadian Institutes of Health Research (CIHR) operating grant to TD. TD is also supported by a Government of Canada Research Chair award, a Canadian Foundation for Innovation grant (CFI-JELF), and an Alberta Innovation and Advanced Education grant.

### Conflict of interest statement

The authors declare that the research was conducted in the absence of any commercial or financial relationships that could be construed as a potential conflict of interest.
